# Unusual Stylar-End Breakdown and Sour Rot on Key Lime (*Citrus aurantiifolia*) in Pre-Harvest Condition in Italy

**DOI:** 10.3390/plants10050989

**Published:** 2021-05-16

**Authors:** Giorgio Gusella, Alberto Fiorenza, Dalia Aiello, Giancarlo Polizzi

**Affiliations:** Dipartimento di Agricoltura, Alimentazione e Ambiente, sez. Patologia Vegetale, University of Catania, Via S. Sofia 100, 95123 Catania, Italy; giorgio.gusella@phd.unict.it (G.G.); alberto.fiorenza.93@gmail.com (A.F.); gpolizzi@unict.it (G.P.)

**Keywords:** stylar-and rot, disorder, *Geotrichum citri-aurantii*, fungal disease, molecular characterization

## Abstract

Key lime (*Citrus aurantiifolia*) is an emerging crop in Italy, especially in the Southern regions, where the environmental conditions are suitable for its cultivation. A field survey in Sicily in a commercial orchard of Key lime revealed the widespread presence of water-soaked spots and sunken/dry lesions at the stylar-end, mainly in pre-harvest condition. Water-soaked spots were attributed to *Geotrichum citri-aurantii*, an agent of sour rot on *Citrus* spp., whereas the sunken/dry lesion was attributed to the physiological disorder known as stylar-end breakdown. Sour rot and stylar-end breakdown are usually considered post-harvest diseases and rarely found in the field on fruit still attached to the tree. Although *Geotrichum citri-aurantii* is not responsible for the stylar-end breakdown, its association with this alteration reveals the importance of the environmental conditions and the agronomic practices in diseases/disorders development. In addition, to our knowledge, this is the first report of *Geotrichum citri-aurantii* on Key lime in Europe.

## 1. Introduction

Lime is a hesperidium fruit (*Rutaceae*) classified in three groups: sweet lime (*Citrus limetta*), acid lime, including the “Key” lime (*C. aurantiifolia*), and the Australian finger lime (*C. australasica*) [1]. Global top producers of lemons/limes (1000 metric tons unit) include Mexico (2870), the European Union (1640) and Argentina (1030) [2]. Many pathogen-pest complexes are known as a severe threat for lime production around the world. Regarding fungal diseases, Anthracnose (*Colletotrichum acutatum*), Melanose (*Diaporthe citri*), Scab (*Elsinoe fawcettii*) and Stem-end rot (*Lasiodiplodia theobromae* and *Phomopsis citri*) are the main diseases reported [3,4,5]. Important post-harvest diseases are caused by *Penicillium digitatum* (green mould), *P. italicum* (blue mould) and *Geotrichum candidum* (sour rot) [4,6,7]. No less important than pathological decays, post-harvest disorders such as chilling injury, oil spotting (oleocellosis) and stylar-end breakdown (SEB) represent a significant limiting factor of lime quality [5,8]. A survey in a commercial Key lime orchard in Sicily (Italy) cultivated under shade netting revealed an abundant presence of fruit (attached to the trees and harvested as well) showing lesions at the stylar-end. Part of the fruit observed in the field showed water-soaked spots at the stylar-end turning from light to dark yellow, slightly raised spots, and sometimes the presence of white mycelium on fruit epicarp. Other fruit showed sunken, dry, tan lesions at the stylar-end, sometimes in conjunction with the other symptoms described above. Since Key lime is considered a new and emerging crop in Italy, especially in the Southern regions, it is crucial to identify pathogens and limiting factors for this crop in order to properly manage the cultivation. The aim of this study was to investigate the etiology of the lesions observed in both phases of pre-harvest as well as post-harvest.

## 2. Results

**Figure 1:**
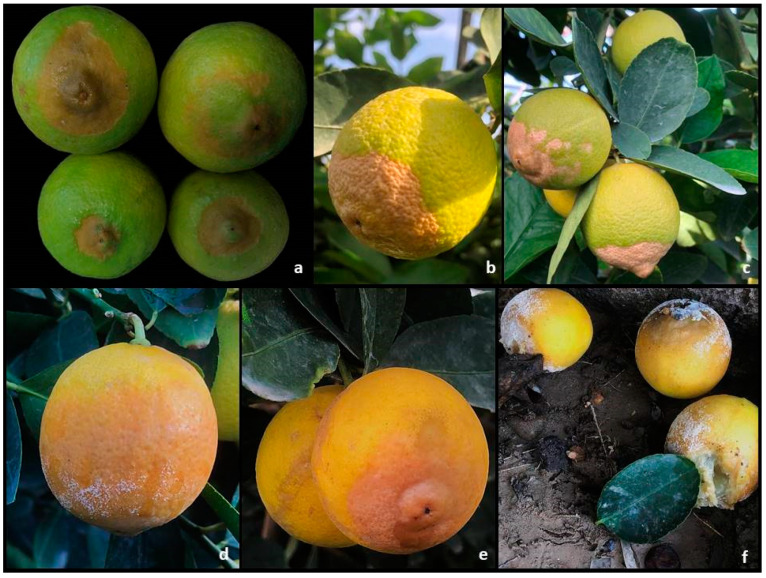
Symptoms on Key lime in pre-harvest. (**a**–**c**) Sunken and dry lesions on Key limes (SEB); (**d**,**e**) symptoms of sour rot; (**f**) rotten limes on the ground covered by abundant white *Geotrichum* mycelium.

**Figure 2:**
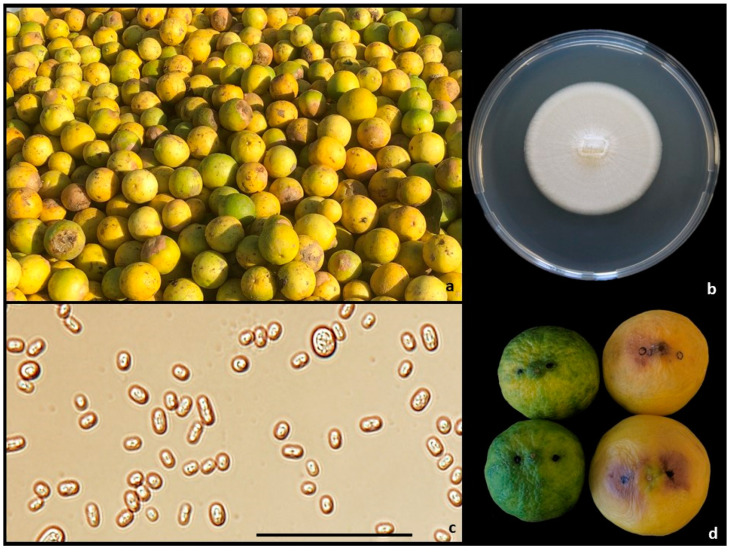
Sour rot details. (**a** Key limes after harvesting showing symptoms at the stylar-end (SEB and sour rot); (**b**) 10-days-old colony of *Geotrichum citri-aurantii* isolate Geo1 on PDA; (**c**) arthroconidia of *G*. *citri-aurantii* isolate Geo1 (scale bar = 50 μm); (**d**) ripe (left) and over-ripe (right) Key limes seven days after inoculation. Black dots represent the inoculation sites.

Field survey conducted in November of 2020 in three hectares of commercial orchard of Key lime (~1300 trees) cultivated under shade netting in Catania Province, Sicily (Italy) showed the presence of almost 15% symptomatic fruit. Limes still attached to the trees, as well as those already harvested, showed sunken and dry lesions at the stylar-end, often covering up to half of the fruit surface (Figure 1a–c). Symptoms of water-soaked spots at the stylar-end were also observed on attached and harvested fruits, often showing white mycelium on the rotten area (Figure 1d–f and Figure 2a). Fruit showing SEB symptoms, once cut in half, showed the presence of translucid areas close to the stylar-end and the mammiform tip. From both kinds of symptoms, a filamentous yeast-like fungus was consistently isolated, having a thin, flat, white-to-cream, lightly powdery mycelium, firstly identified as *Geotrichum*-like (Figure 2b). Arthroconidia were hyaline, sub-globose-to-cylindrical in shape, ranging from (5.4-) 7.2 ± 1.1 (-10.4) × (3-) 4 ± 0.6 (-5.2) μm (Figure 2c). Regarding the molecular characterization, chromatograms resulting from the sequencing of partial internal transcriber spacer region (ITS) revealed the presence of dual peaks, especially in forwards. Multiple attempts were made in isolates’ reculturing, DNA re-extractions and sequencing, but the amplicons always showed dual peaks, which was the reason why the BLAST search was conducted using the reverse sequences only, which seemed to be clean for at least 230 bp. Edited reverse amplicons (232 bp) showed 100% identity with *Galactomyces citri-aurantii* (asexual morph *Geotrichum citri-aurantii*) (GenBank accession MH153586). The results from the pathogenicity test, summarized in Table 1, reveal that only over-ripe limes incubated at 25 °C developed symptoms of water-soaked spots at 48 h (22%) and seven days (77%) after inoculation, as well as ripe lemons “Femminello Siracusano 2 KR” after seven days (55%) (Figure 2d). Some fruit developed white mycelium on the epicarp. None of the inoculated fruit developed sunken/dry lesions, typical symptom of SEB, at the stylar-end. Therefore, this result induced us to attribute this symptom to the physiological disorder known as stylar-end breakdown (SEB), whereas the water-soaked spots were attributed to the sour rot. Controls did not show any symptom. Reisolations from symptomatic limes and lemons showed 100% *G. citri-aurantii* and no fungal colonies from controls.

## 3. Discussion

The results of the present study highlight unusual alterations of Key lime in pre-harvest condition. Field surveys revealed two different symptoms at the stylar-end: water-soaked spots covered with a yeasty, sometimes wrinkled layer of white-colored mycelium, and sunken/dry lesions. The results of isolations showed a high incidence of a yeast-fungus, characterized as *G. citri-aurantii*. The identity of the six isolates characterized based on morphological aspects was complemented by means of the partial internal transcribed spacer sequences. Difficulties in the sequencing of the ITS region, and the constant presence of dual peaks in the resulting amplicons after many sequencing efforts, emerged from this study. Other authors reported the same issues with *Geotrichum* spp., especially for the ribosomial DNA, which seemed to be characterized by a high heterogeneity [9,10,11]. These authors constantly noticed the presence of dual peaks, and most of the analyzed strains were processed multiple times in order to avoid the possibility of some methodological mistake. The results of previous researches demonstrated the presence of many different ITS1-5.8S-ITS2 variants within the same strains [9,10,11]. The similarity of our difficult sequencing of the ribosomial DNA led us to hypothesize intragenomic rDNA variability within our isolates, as previously confirmed [9,10,11]. Sour rot of citrus fruit was described in California in 1917 and was attributed to *Oospora citri-aurantii* [12], nowadays named *G. citri-aurantii* [13], a variety of *G. candidum* (Mycobank current name *G. candidum* var. *citri-aurantii* MB# 123736). Sour rot usually leads to a complete disintegration of the fruit due to the degrading activity of the extracellular enzymes of the fungus in the rind, segment walls and juice vesicles [13]. The physiological disorder known as stylar-end breakdown has been deeply investigated, being one of the main limiting factors in lime production and commercialization. It is mainly considered a post-harvest disorder, but it occasionally occurs in pre-harvest under certain conditions: particularly with high temperatures after rainy events [13]. Suffice it to say that the lime industry in the US has deemed SEB its number one problem [14]. For many years, researchers thought that it was a rind disorder determined by a chain reaction of cellular breakdown that rapidly spread to the albedo and flavedo after the fruit struck the ground [14]. Many studies investigated the causes of this disorder, leading to the conclusion that the affected area was determined by the juice vesicles’ rupture and chlorophyll destruction in the rind [13]. Further investigations revealed that the cause of the characteristic symptom at the stylar-end was the rupture of the juice vesicles and the passing of the juice into the rind [14]. Although there is no evidence of any microorganism responsible for SEB, but only physiological processes, it is interesting to underline the environmental conditions responsible for its occurrence and the possible association with some microorganism. The results of our pathogenicity tests revealed that *G. citri-aurantii* was responsible for the water-soaked spots at the stylar-end. Devenport et al. [14] studied three factors that could contribute to the incidence of SEB, which were bruising, fruit maturity (size) and field heat, and tried to elucidate the mechanisms involved in the vesicles’ rupture. One of the mechanisms could pertain to the weakening of vesicles’ membranes and cell walls, and the consequent inability to withstand the turgor pressure; and another mechanism could pertain to the resistance limit point of the turgor and/or the internal fruit pressure. The authors concluded that excessive turgor pressure and heat stress associated with fruit maturity were the causes of the disorder; thereby, they strongly recommend that one avoid over-ripening on the tree [14]. It is interesting to note that all these factors that are related to SEB disorder are the same that predispose limes to sour rot. As demonstrated for lemon fruit, the physiological age, storage time and water status of the fruit are the main factors influencing susceptibility to sour rot [15]. Although we cannot affirm any relation of causality between the presence of *G. citri-aurantii* in fruit affected by SEB, we can indeed confirm, as previously affirmed [16], that fruit showing SEB symptoms facilitate the development of the sour rot. Our field survey revealed that lime fruit showing sour rot in pre-harvest were significantly over-ripe. Pathogenicity tests, in fact, confirmed that the highest percentage of disease incidence resulted in over-ripe limes incubated at 25 °C, whereas over-ripe limes incubated at 4 °C and ripe (green) limes incubated at 25 °C did not show any symptoms of sour rot. These results indicate that environmental and physiological factors are crucial in the disease development. Harvesting practices therefore become an important step in order to control SEB disorder and sour rot. The orchard investigated in our study showed different agronomic and environmental conditions important in fruit diseases/alterations. Trees were grown under shade netting, and this, with respect to an open-air system, could provide suitable conditions for the pathogen in terms of humidity and canopy ventilation, along with the heavy clay/lime soil and the presence of infected fruit left in the orchard. As demonstrated in California, where environmental conditions for sour rot of peach and nectarine infrequently occur, a high soil pathogen population, the presence of fallen fruit remaining on the ground and episodes of high humidity led to sour rot in the field [17]. Our field observations revealed the presence of infected fruit that remained on the ground or attached to the trees. This condition, in addition to the presence of rain or insects, represents a critical situation for the growers. The dispersal of inoculum vectored by insects is reported for this pathogen, mainly carried on the body surface of fruit flies and nitidulid beetles [17], and therefore it is strongly recommended that one discard infected fruit from the orchard. *Geotrichum candidum* is widely reported to be found in the soil [18,19], and this represents an important source of inoculum, especially in the case of soil contamination of the fruit packing line from the orchard to the packinghouse [17]. Investigating the *Geotrichum* spp. soil population is relevant in terms of its management. Studies conducted in California demonstrated a decline of the *G. candidum* population with an increasing soil depth [17]. Both sour rot and SEB are considered mainly post-harvest diseases. The results of our study highlight how environmental and agronomic conditions are very important in preventing important diseases and/or alterations.

## 4. Materials and Methods

### 4.1. Isolation

The incidence of symptomatic fruit in field was determined on approximately 40 plants, randomly selected. Fifty symptomatic fruit samples were brought to the Plant Pathology laboratory of the Dipartimento di Agricoltura, Alimentazione e Ambiente, Sezione di Patologia vegetale, University of Catania for further investigations. Small sections (0.5 × 0.5 cm^2^) of diseased albedo and flavedo tissues were surface-disinfected for 1 min in 1.5% sodium hypochlorite, rinsed in sterile water, placed on potato dextrose agar (PDA, Lickson) amended with 100 mg/liter of streptomycin sulfate (Sigma-Aldrich, St. Louis, MO, USA) to prevent bacterial growth, and then incubated at 25 ± 1 °C for three–four days. Representative single-spore isolates of fungal colonies were obtained from pure cultures grown on PDA at 25 ± 1 °C.

### 4.2. Morphological and Molecular Characterization

For the morphological characterization of the pathogen, the length and width of 30 arthroconidia from the seven-days-old colony of the isolate Geo1 grown on PDA were measured using a fluorescence microscope (Olympus-BX61) coupled to an Olympus DP70 digital camera; images and measurements were captured using the software analySIS Image Processing. Dimensions are reported as the minimum and maximum in parentheses, and the average is reported with the standard deviation. Representative isolates were stored in the Plant Pathology collection of the Dipartimento di Agricoltura, Alimentazione e Ambiente, Sezione di Patologia vegetale, University of Catania. Genomic DNA of the selected isolates (Geo1, 2, 5, 8, 9 and 11) was extracted using the Gentra Puregene Yeast/Bact. Kit (Qiagen), Wizard Genomic DNA Purification Kit (Promega Corporation, Madison, WI, USA), and also directly extracted by Macrogen Inc. (Seoul, South Korea). The internal transcriber spacer region (ITS) of the nuclear ribosomal RNA cluster was repeatedly amplified with different primer combinations, using ITS1f/ITS5 and ITS4 [20,21]. PCR amplification conditions were set as follows: initial denaturation temperature of 94 °C for 30 s, followed by 35 cycles at the denaturation temperature of 94 °C for 30 s, annealing temperature of 50–52 °C for 1 min, extension at 68 °C for 1 min, and final extension at 68 °C for 5 min. PCR products were purified and sequenced in both directions by Macrogen Inc. (South Korea). The same region was also sequenced by Macrogen Inc. (South Korea) using primers ITS1/ITS4 [21]. Sequences were read and edited using MEGAX: Molecular Evolutionary Genetics Analysis [22]. BLAST searches were performed against the NCBI nucleotide database [23].

### 4.3. Pathogenicity Test

In order to fulfil Koch’s postulates, a total of 18 fruit for each treatment condition were used in the pathogenicity tests. Two inoculation sites were used for each fruit at the stylar-end. Five fruit for each treatment were used as control. Treatment conditions consisted of: (a) over-ripe Key limes; (b) ripe lemons “Femminello Siracusano 2 KR”; and (c) ripe (green) Key limes. Each treatment was incubated at 25 °C, and treatments a and b were also incubated at 4 °C. Fruit were surface-disinfected in 2% sodium hypochlorite solution for 10 min and rinsed twice in sterile deionized water. Once completely air-dried on a laboratory bench, two wounds were made with a needle (insulin syringe) at the stylar-end on the opposite sides respectively, and 20 μL of 10^6^ arthroconidia/mL suspension of the isolate Geo1 were pipetted onto two inoculation sites. Controls consisted of wounded fruit inoculated with sterile water only. Replicates were kept in plastic containers to maintain a high humidity in a growth chamber with a 12 h photoperiod at 25 ± 1 °C and in the refrigerator at 4 °C. The disease incidence, indicated as the percentage (%) of fruit showing a rotten area in at least one inoculation site, was recorded 48 h and seven days after inoculation. Reisolations were conducted as described above from representative inoculated fruit and from controls in order to fulfill Koch’s postulates.

## 5. Conclusions

This study underlines an unusual presence in pre-harvest of stylar-end breakdown and sour rot caused by *G*. *citri-aurantii* on Key lime, usually considered post-harvest diseases. Environmental conditions and agronomic practices could be crucial to prevent the occurrence of both alterations on this crop, which represents an important economic income for growers in Italy and Mediterranean countries. In addition, to our knowledge, this is the first report of *G*. *citri-aurantii* on Key lime in Europe.

## Figures and Tables

**Table 1:** Treatment conditions and Disease Incidence (D. I.) in pathogenicity test.

Treatment Conditions	D.I. 48 h	D.I. 7 Days
Over-ripe lime 25 °C	22%	77%
Ripe (green) lime 25 °C	0%	0%
Over-ripe lime 4 °C	0%	0%
Ripe lemon 25 °C	0%	55%
Ripe lemon 4 °C	0%	0%
Control	0%	0%

## Data Availability

Data available on request due to restrictions, e.g., privacy or ethical.
